# The Earth’s Population Can Reach 14 Billion in the 23rd Century without Significant Adverse Effects on Survivability

**DOI:** 10.3390/ijerph14080885

**Published:** 2017-08-07

**Authors:** Vladimir F. Krapivin, Costas A. Varotsos, Vladimir Yu. Soldatov

**Affiliations:** 1 Kotelnikov Institute of Radio engineering and Electronics, Russian Academy of Sciences, Moscow 125009, Russian Federation; vkrapivin_36@mail.ru (V.F.K.); soldatov_v@list.ru (V.Y.S.); 2 Department of Environmental Physics and Meteorology, Faculty of Physics, National and Kapodistrian University of Athens, Athens 157 72, Greece

**Keywords:** climate models, ocean, soil-plant formation, survivability-biocomplexity, biochemical cycle

## Abstract

This paper presents the results obtained from the study of the sustainable state between nature and human society on a global scale, focusing on the most critical interactions between the natural and anthropogenic processes. Apart from the conventional global models, the basic tool employed herein is the newly proposed complex model entitled “nature-society system (NSS) model”, through which a reliable modeling of the processes taking place in the global climate-nature-society system (CNSS) is achieved. This universal tool is mainly based on the information technology that allows the adaptive conformance of the parametric and functional space of this model. The structure of this model includes the global biogeochemical cycles, the hydrological cycle, the demographic processes and a simple climate model. In this model, the survivability indicator is used as a criterion for the survival of humanity, which defines a trend in the dynamics of the total biomass of the biosphere, taking into account the trends of the biocomplexity dynamics of the land and hydrosphere ecosystems. It should be stressed that there are no other complex global models comparable to those of the CNSS model developed here. The potential of this global model is demonstrated through specific examples in which the classification of the terrestrial ecosystem is accomplished by separating 30 soil-plant formations for geographic pixels 4° × 5°. In addition, humanity is considered to be represented by three groups of economic development status (high, transition, developing) and the World Ocean is parameterized by three latitude zones (low, middle, high). The modelling results obtained show the dynamics of the CNSS at the beginning of the 23rd century, according to which the world population can reach the level of 14 billion without the occurrence of major negative impacts.

## 1. Introduction

Nowadays, the environmental impacts of human activities have expanded to a large spatial scale and have become more rapid [1,2]. Initially, these activities transformed places or areas, while today they are transforming almost all of Earth [3]. Changes that have taken place in decades or centuries are now happening in a few years. This is due to the fact that atmospheric and climatic processes obey non-linear dynamics [4,5,6,7,8].

The problem of the sustainable development of human society has not been alleviated. On the contrary, it has been strengthened in the 21st century. If human society had been actually on the brink of nuclear war in the mid-20th century (when there was a crisis in the Caribbean), the climate-nature-society system (CNSS) would now be in critical condition for a number of reasons, such as:

- The premature increase in the world population compared to the increase in productivity of agricultural and natural ecosystems would lead to a decrease in the volume of food per capita. The food deficit is a fact in many areas. Food per person decreases over time and an increase in hungry people is expected [9].
- The environmental response to anthropogenic intervention to natural cycles would be manifested by the intensification of natural disasters, including the emergence of new incurable diseases [10,11].
- Global climate change due to the disturbance of cycles of greenhouse gases and water resources leads to a modification of spatial distribution of water resources, including drinking water [12].
- The development of new powerful weapons would contribute additional uncertainties in the problem of human population survivability [13].
- The intensification of both international and regional conflicts would be followed by dramatic changes in the globalization and decentralization processes which would not encourage the improvement of the living conditions of the population [14].
- There would be ecological consequences of mobile communication media including mobile phones [15].

Under these circumstances, the strained global relations would have as result to put aside the solution of the survivability problem. which is impossible on a regional scale. It is necessary to develop an information technology that allows a comprehensive description of the global ecological, demographic, social-economic and climatic processes that take place in the CNSS. This technique would allow to search for constructive strategies for the CNSS survivability taking into account existing assessments and forecasts of environmental resources. A cornerstone of the concept of sustainable co-existence of nature and humans is the convention that all countries should seek appropriate strategies for the evolution of the biosphere-population system, taking into account the reserves of the biosphere. The global population in its tendency to the reduction of poverty must realise that the reserves of biosphere are exhaustible. Therefore, the complex objectives of the global population must be research and monitoring related to conservation and sustainability. As for this problem, there are many investigations based on global models [16,17,18,19,20,21,22,23]. These and others studies of global environmental processes are based on different models of the present view of the CNSS structure. Many of them have a virtual character based on the philosophy-ideology of the world state. The constructive approach to the global environmental modeling was proposed by Moisseev [24] who formulated a well-defined conceptual model for the biosphere that differs greatly from the known global models of the Club of Rome [25,26,27,28,29]. After many researches a credible mathematical approach to the global environmental model was finally developed, and provided simulation experiments with global environmental processes including assessments of the effects of anthropogenic impacts on biosphere ecosystems [30,31,32,33,34].

The difference between the models of the Club of Rome and the other models lies mainly in the following methodological principles [35]:

- The authors of the models of the Club of Rome focused their main attention both on global economic processes which connect to separate environmental processes and secondly selecting the demographic block as a key element of the global model.
- Moisseev’s [24] starting position was the research of the biosphere considering the human as an element of the biosphere and that the demographic and economic processes are only taken into account in the systematic analysis of the global ecological evolution.

The present socio-economic theories of sustainable development are far from Moisseev’s ideas and certainly from Vernadsky’s noosphere theory [36]. Many indicators such as Happy Planet Index (HPI), Human development Index (HDI), Food Production Index (FPI), Gross Domestic Product (GDP) and others undoubtedly help to assess the development tendencies in a particular CNSS section but have difficulties in the complex evaluation of the CNSS evolution. It is possible only by using a global model that allows to taking into account the maximum number of direct and indirect couplings present in the CNSS.

The trend towards improving global models is characterized by efforts to improve their precision and reduce the provision of information requirements. At the same time the complexity of organized reality prevents this approach of improvement and brings a set of constraints associated with chaotic environmental processes and the multidimensional problem [32,37,38,39,40,41]. Indeed, each global model has an individual character and focuses on a limited set of environmental processes and elements. Krapivin et al. [42,43] proposed a new approach to the development of a global model based on the use of high-level tools for the utilization of separate operations associated with the description of processes in the CNSS. In particular, the geoecological information-modeling system (GIMS) was developed whose architecture is based on the combined use of GIS-technology and modeling tools.

This paper proposes the use of GIMS as a universal tool for the complex parameterization of the most important global processes for the investigation of a sustainable state between nature and human society, taking into account existing global models that describe different processes in the CNSS [43,44,45]. The GIMS/CNSS consists of many mechanisms, which operate autonomously to represent a part of the desired functionality. Therefore, the architecture of the complex GIMS/CNSS Global Model was developed in such a way to demonstrate an integrated pattern of direct and indirect relationships between the traditional processes in the CNSS.

## 2. General Description of the GIMS/CNSS Model

Key aspect of the assessment of the humanity survivability is the ecological status of the natural evolution of the Earth, which determines food production and other conditions already said. Certainly, the level of self-organization and the structure of the CNSS depends on many factors of the co-evolution of nature-population as elements of the biosphere. Consequently, the composition of the CNSS model is only possible by a synergistic approach that dictates the form and structure of the GIMS/CNSS. GIMS plays a management role by providing coordination between CNSS components and expanding their operations.

Following this approach, the key components of the GIMS/CNSS are defined as the information core for ecological, geophysical, hydrological, biocenotic and demographic processes taking place across the globe. The Earth’s surface Ξ is divided into World Ocean Ξ*_O_* and the land Ξ*_L_* (Ξ = Ξ*_L_*∪Ξ*_O_*). The land surface Ξ*_L_* is covered by a geographical grid with discrete steps of Δ*ϕ_i_* and Δ*λ_j_* of latitude and longitude, respectively, so that all processes within the pixel Ξ*_Lij_* = {(*ϕ*, *λ*): *ϕ_i_* ≤ *ϕ* ≤ *ϕ_i_* + Δ*ϕ_i_*; *λ_j_* ≤ *λ* ≤ *λ_j_* + Δ*λ_j_*} are considered uniform and parameterized by the point models. Each pixel area σ*_ij_* = χ*_ϕ_*χ*_λ_*Δ*ϕ_i_*Δ*λ_j_* is occupied by the soil-plant formation (*r*_1_th part), the agricultural vegetation (*r*_2_th part), the hydrophysical objects (*r*_3_th part), and the anthropogenic objects ((1−*r*_1_−*r*_2_−*r*_3_)th part), where χ*_ϕ_*(≈111 km) and χ*_λ_*(=111.3 cos *ϕ*) are the number of kilometers to a degree of latitude and longitude, respectively.

In the case of the World Ocean, three latitudinal zones are separated: the equatorial zone Ξ*_O_*_1_ = {(*ϕ*, *λ*): *ϕ*∈[0° N, 30° N]∪[0° S, 30° S]; 0° ≤ *λ* ≤ 360°}, temperate latitudes Ξ*_O_*_2_ = {(*ϕ*, *λ*): *ϕ*∈[30° N, 60° N]∪[30°S, 60°S]; 0° ≤ *λ* ≤ 360°} and Arctic and Antarctic zone Ξ*_O_*_3_ = {(*ϕ*, *λ*): *ϕ*∈[60° N, 90° N]∪[60° S, 90° S]; 0° ≤ *λ* ≤ 360°}. Pelagic Ξ_O1P_ and upwelling Ξ_O1U_ aquatories are selected in the Ξ_O1_ zone to differ in productivity and gas exchange rate on the air-water boundary [46,47].

**Figure 1:**
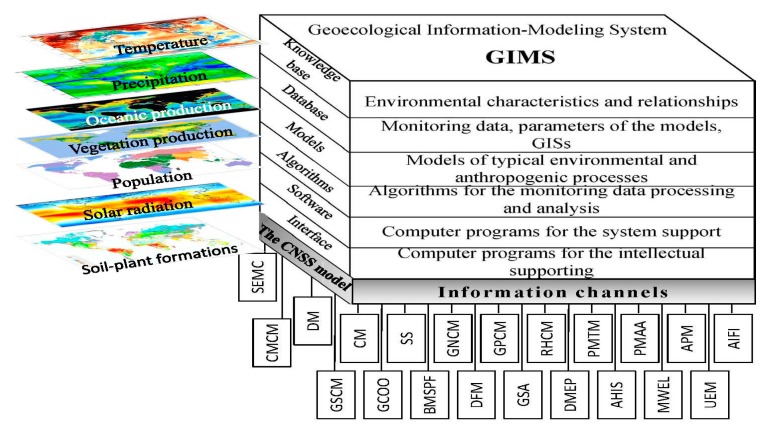
The GIMS/CNSS block-diagram. Abbreviation expansion is given in Table 1.

Figure 1 and Table 1 show the GIMS/CNSS block structure that is synthesized by taking into account the components and parameters of the global bio-geosystem, managed by geoinformatics monitoring systems. The spatial structure of GIMS/CNSS is defined by the available database and knowledge base. The simplest version of the point model is made when the World Ocean and land are considered as unique element of the planet. The spatial heterogeneity is carried out by the various forms of global space sampling. A basic form of spatial digitization is the choice of a uniform grid Δ*ϕ* × Δ*λ*. The GIMS allows the different spatial grids for each CNSS model item that supports the integration of pixels Ξ*_Lij_*. This kind of spatial structure of the biosphere allows the model to be adapted to the heterogeneities of the databases and to perform simulation experiments with the realization of the individual regions.

Depending on the peculiarities of the natural process under consideration, a regional structure can be identified with the climatic and geographic zones, the continents, the natural bio-forms and the socio-administrative structures. For example, Krapivin and Vilkova [48] divided the land’s biosphere into the pixels of magnitude Δ*ϕ* = 4° and Δ*λ* = 5°. In more details, the biogeocenotic processes are studied in Δ*ϕ* = Δ*λ* = 0.5° [31]; the socio-economic processes are usually represented by three or nine regions, according to the status of the country development [49]; the atmospheric processes in biogeochemical cycles of long-living elements are approached with the point models [50,51]; the functioning of the oceanic ecosystems are represented by the heterogeneous spatial structure including pixels Ξ_O*ij*_ of shelf zone and pelagic zones of four oceans [46]. Tarko [52] developed the Moscow Global Biosphere Model, where the World Ocean is represented by the upper quasi-uniform and deep layers separately for four latitudinal zones in the north and south aquatories. It is emphasized that GIMS allows for the combined use of these parameterizations.

## 3. Description of the GIMS/CNSS Items

The GIMS/CNSS items listed in Table 1 perform the calculations of the energy and matter flows between the spatial digitization pixels of the biosphere taking into account its components. The GIMS/CNSS stability is provided by the information channels linking the functional items so that the change or modification of the item does not affect other items.

Item GSA provides the symbol-parametric identification for pixel components including the soil-plant formations, pollutant sources, water ecosystems and population. As a result, the matrix structures are formed as spatial identifiers of the CNSS elements. Item AHIS focuses on the task of the assessment of the survivability level of population based on available indicators. One of these is the survivability indicator:

(1) $$J(t)=\frac{1}{\sigma }\left\{\sum_{(i,j)\in \Xi_{L}}\sigma_{ij}\left[r_{1}\frac{R_{\Phi }^{1}(i,j,t)}{R_{\Phi }^{1}(i,j,t_{0})}+r_{2}\frac{R_{\Phi }^{2}(i,j,t)}{R_{\Phi }^{2}(i,j,t_{0})}+r_{3}\frac{R_{\Phi }^{3}(i,j,t)}{R_{\Phi }^{3}(i,j,t_{0})}\right]+\sum_{s=1}^{3}\sigma_{Os}\frac{R_{P}(s,t)}{R_{P}(s,t_{0})}\right\}$$

where

$$\begin{array}{l} R_{\Phi }^{k}\left(i,j,t\right)=R_{\Phi }^{*k}\left(i,j\right)\min\left\{a_{C}\frac{C_{A}(t)-\Gamma (i)}{b_{C}+C_{A}(t)+\Gamma (i)},\;\frac{a_{E}E(i,t)}{b_{E}+E(i,t)},\;\frac{a_{W}W\left(i,j,t\right)}{b_{W}+W\left(i,j,t\right)},R_{T}\left(i,t\right)\right\}+M_{\Phi }^{k}\left(i,j,t\right)\\ R_{T}\left(i,t\right)=\max\left\{0,\;\frac{T\left(i,t\right)-T_{\min}(\kappa )}{T_{opt}\left(\kappa \right)-T_{\min}\left(\kappa \right)}\exp\left[a_{T}-b_{T}\frac{T\left(i,t\right)-T_{\min}\left(\kappa \right)}{T_{opt}\left(\kappa \right)-T_{\min}\left(\kappa \right)}\right]\right\},\\ M_{\Phi }^{k}\left(i,j,t\right)=K_{B}\Phi^{*k}\left(i,j\right)\max\left\{0,\;\frac{\rho_{T}T\left(i,t\right)}{d_{T}+T\left(i,t\right)},\;\frac{d_{a}W\left(i,j,t\right)}{d_{b}+W\left(i,j,t\right)}\right\},\\ T\left(i,t\right)=T_{g}(t)+\left(T_{N}(t)-T_{e}(t)\right)\left(\sin^{2}\phi_{T}-\sin^{2}(4i)\right),\\ R_{P}\left(s,t\right)=R_{P}^{*s}\min\left\{\Upsilon_{0}\left(T_{W}\right),\;\Upsilon_{1}\left(E\right),\;\Upsilon_{2}\left(n\right),\;\Upsilon_{3}\left(P\right)\right\},\;s=1,2,3,\\ \Upsilon_{0}\left(T_{W}\right)=\frac{T_{W}}{T_{Wopt}}\exp\left[\theta_{W}\left(1-\frac{T_{W}}{T_{Wopt}}\right)\right],\;\Upsilon_{1}\left(E\right)=\frac{E}{E_{\max}}\exp\left[\theta_{E}\left(1-\frac{E}{E_{\max}}\right)\right],\\ \Upsilon_{2}\left(n\right)={\left[1-\exp\left\{-\gamma_{n}\frac{n\left(s,t\right)}{n\left(s,t_{0}\right)}\right\}\right]}^{\theta_{n}},\;\Upsilon_{3}\left(P\right)=1-\exp\left\{-\gamma_{P}\frac{P\left(s,t\right)}{P\left(s,t_{0}\right)}\right\}, \end{array}$$

Table 2 lists the model parameters determined by taking into account the data of separate models from Table 1 and minimizing the disagreement between the pre-historic trends of CO_2_, the global population size and the relevant model results during 2000–2015. A comparative analysis of the results of the model and of the global temperature trend over the period 2000–2015 showed credibility of the appropriate results at the level of 7–10%.

*C_A_* is the CO_2_ content in the atmosphere (ppmv), *E* is the solar radiation (W/m^2^), *W* is the precipitation (mm/year), *T_N_* and *T_e_* are the global temperatures at the pole and equator, respectively (°C); *T*_g_ is the global average temperature (°C); *T*_min_ and *T*_opt_ are the critical and optimal temperatures for photosynthesis (°C), respectively [63,64]; *ϕ_T_* is the latitude at which *T*(*i*,*t*) = *T_g_*; *E*_max_ is the solar radiation corresponding to maximal photosynthesis; *n* is the content of the biogenic salts (mg/m^2^); *P* is the phytoplankton biomass (mg/m^2^); *t*_0_(2015) is the starting time, when global average production is assessed by $R_{\Phi }^{*}$ (2015) = 48.7 PgC/year and $R_{P}^{*}$ (2015) = 56.2 PgC/year. Under this $R_{P}^{*1}=0.049\;PgC/\mathrm{day}$ in Ξ*_O_*_3_, $R_{P}^{*2}=0.033\;PgC/\mathrm{day}$ in Ξ*_O_*_1_, and $R_{P}^{*3}=0.072\;PgC/\mathrm{day}$ in Ξ*_O_*_3_ [65,66,67].

Indicator *J*(*t*) is an integral feature of the CNSS complexity that reflects the individuality of its structure and its evolution at the time *t*. According to the laws of natural evolution the decrease or increase of *J*(*t*) will reflect the ability of C NSS to survive. Moreover, the reduction of *J*(*t*) corresponds to the negative disturbance of the biogeochemical cycles that intensify resource-depletion processes and shift the vector of energetic exchange between the basic functions of the CNSS. In particular, the reduction in *J*(*t*) leads to a reduction in total food reserves which may be reflected by the food production index (FDI) which is a function of climate, scientific-technical progress and economic factors [68].

The item CM provides the calculation of the spatial distribution of the mean annual temperature of the atmosphere based on the simple climate model developed by Mintzer [54] and modified by Krapivin et al. [42] as:

Δ*T_g_* = Δ*T*_CO2_ + Δ*T*_N2O_ + Δ*T*_CH4_ + Δ*T*_O3_ + ΔT_CFC11_ + ΔT_CFC12_, *T*(*ϕ*) = *T_g_* + *γ*(*sin*^2^*ϕ_T_* − *sin*^2^*ϕ*), (2)

where *γ* is the difference of atmospheric temperatures between the pole and equator, *ϕ_T_* is the latitude, where *T*(*ϕ*) = *T_g_*,

Δ*T*_CO2_ = −0.677 + 3.019*ln*[*C_А_*(*t*)/*C_А_*(t*)], Δ*T*_N2O_ = 0.057[N_2_O(*t*)^1/2^ − N_2_O(t*)^1/2^], Δ*T*_CH4_ = 0.019[CH_4_(*t*)^1/2^ − CH_4_(t*)^1/2^], Δ*T*_O3_ = 0.7[O_3_(*t*) − O_3_(t*)]/15, ΔT_CFC11_ = 0.14[CFC11(*t*) − CFC11(t*)], ΔT_CFC12_ = 0.16[CFC12(*t*) − CFC12(t*)]. (3)

The value of t* is identified by the year 1980, when the GHG concentration were known (CO_2_ 337.7 ppmv; N_2_O 270 ppb; CH_4_ 722 ppb; CFC11 167.99 ppb; CFC12 307.75 ppb). Items CMCM, GNCM and GCOO calculate concentrations of *C_А_*(*t*), N_2_O(*t*), CH_4_(*t*), O_3_(*t*) using the corresponding models and CFC11(*t*), and CFC12(*t*) taking into account data provided by Butler and Montzka [69].

The item DM refers to the development of a model of population dynamics *G*(*I*,*j*,*t*) taking into account the environmental factors:

*dG*(*I*,*j*,*t*)/*dt* = *R_G_*(*I*,*j*,*t*) − *M_G_*(*I*,*j*,*t*), (4)

where *R_G_* and *M_G_* are the indicators of birth rate and mortality, respectively. Birth rate and mortality are mainly functions of the food supply and environmental characteristics. Detailed description of these functions is given in [53].

According to [53] the functions *R_G_*(*I*,*j*,*t*) and *M_G_*(*I*,*j*,*t*) in (4) are linked to each other with the following equations:

*R_G_*(*I*,*j*,*t*) = μ*_B_G*(*I*,*j*,*t*), (5)

Where μ*_B_* and μ*_d_* are the coefficients characterizing the birth rate and mortality, respectively; ϖ is the index of the influence of the population density on mortality. These coefficients are functions of environmental and anthropogenic characteristics, notably:

μ*_B_* = ρmin{μ_1_(1 − HDI) + μ_2_HDI; μ_1_(1 − HPI) + μ_2_HPI; μ_1_exp[−ξ_1_FPI/FPI(*t*_0_)] + μ_2_[1 − exp{−ξ_1_FPI/FPI(*t*_0_)}; μ_1_exp[−ξ_2_GDP/GDP(*t*_0_)] + μ_2_[1 − exp{−ξ_2_GDP/GDP(*t*_0_)}; μ_1_exp[−ξ_3_*V_G_*] + μ_2_(1 − exp[−ξ_3_*V_G_*])} (6)

μ*_d_* = *β*min{η_1_(1 − HDI) + η_2_HDI; η_1_(1 − HPI) + η_2_HPI; η_1_exp[−χ_1_FPI/FPI(*t*_0_)] + η_2_[1 − exp{−χ_1_FPI/FPI(*t*_0_)}; η_1_exp[−χ_2_GDP/GDP(*t*_0_)] + η_2_[1 − exp{−χ_2_GDP/GDP(*t*_0_)]}; η_1_exp[−χ_3_*V_G_*] + η_2_(1 − exp[−χ_3_*V_G_*])} (7)

where μ_1_ and μ_2_ are coefficients of maximal and minimal birth rates, respectively; η_1_ and η_2_ are maximal and minimal mortalities, respectively; ρ, *β*, χ_1_, χ_2_, χ_3_, ξ_1_, ξ_2_ and ξ_3_ are adaptation coefficients; *V_G_* is the efficient food amount that is defined as weighed sum of the components of personal food spectrum (calculated by the items UEM, PMAA, MWEL, and PMTM).

In the common case we have:

$$V_{G}(t)=\left\{\sum_{(i,j)\in \Xi_{L}}\sigma_{ij}\left[r_{1}d_{1}R_{\Phi }^{1}(i,j,t)+r_{2}d_{2}R_{\Phi }^{2}(i,j,t)++r_{3}d_{3}R_{\Phi }^{3}(i,j,t)\right]+d_{4}\sum_{s=1}^{3}R_{P}(s,t)\right\}/\sum_{(i,j)\in \Xi_{L}}\sigma_{ij}G(i,j,t)$$

where *d*_1_ (0.023), *d*_2_ (0.65), *d*_3_ (0.11)and *d*_4_ (0.013) are coefficients determining the contribution of the production of the natural vegetation, agricultural plants, land water systems and oceans, to the population food spectrum, respectively.

Each pixel Ξ*_ij_* is characterized by the biocomplexity level and participates in the food production as an element of restricted area that can consolidate different biomes, ecosystems and anthropogenic territories. In order to determine the typical description of the spatial structure of CNSS, the following three socio-economic groups of countries are selected to be represented by respective areas of the land Ξ*_L_*:

- Ξ*_LD_* the area occupied by countries with HDI ∈ [0.85,1]
- Ξ*_LM_* the area occupied by the countries with transition economy (HDI ∈ (0.65,0.85)), and
- Ξ*_LG_* correspond to the territory of the developing countries (HDI ∈ [0,0.65]).

Social costs, economic growth, food insecurity, and environmental disruption in each territory are presented with different intensity. The food supply is made from the following available sources:

- Agricultural technologies are the main food producers that can promote food safety and nutrition security. Global agriculture supplies 2940 kcal per person at present with a forecast of up to 3050 in 2030. Existing protein support per person is estimated at 60 g a day when the medical standard is 70 g. The total protein deficit is estimated at 10 to 25 million tons. Nearly half of the world’s population (7.5 billion) suffers from a lack of protein [70].
- The second major source of the food is fishing and cultivation of fish in natural lakes and reservoirs. In 2016 each person consumed about 22 kg of fish production. At present, the ecosystems of the World Ocean and the seas provide about 20% of the world’s needs for proteins of animal origin. Mainly, oceanic biomass is estimated around 150 thousands of the animal species and 10 thousands of the water-plants with a total weight of about 35 billion tons which is sufficient to survive 35 billion people [71].
- Natural plants and forest in the first series can be considered hypothetical sources of food including wild animals and edible plants, hazelnuts, etc. Further development of the food industry and corresponding science allows the expansion of primary use of natural biomass for food production.

**Figure 2:**
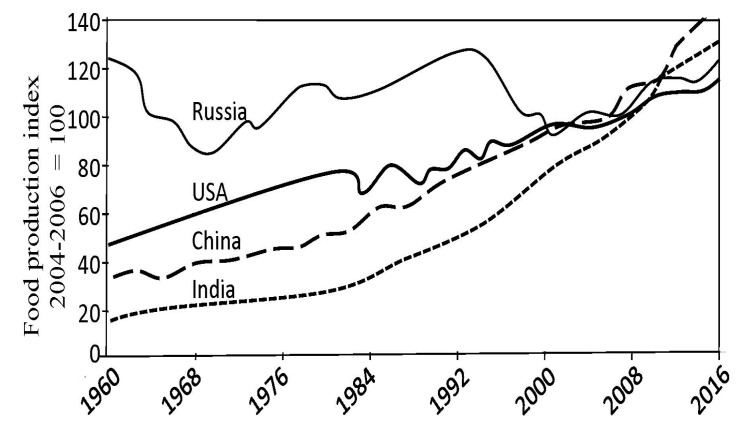
Food production indicators in major countries.

**Figure 3:**
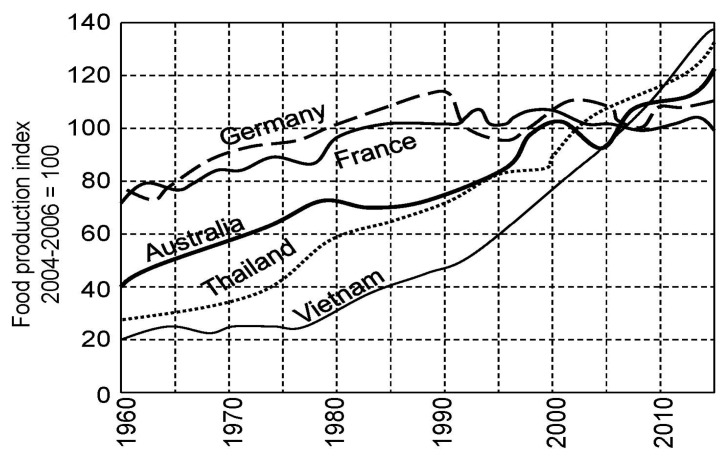
Comparison of food production indicators for developed and developing countries.

**Figure 4:**
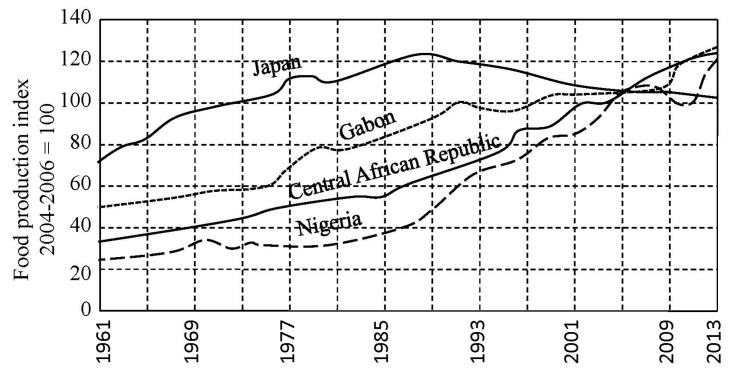
Comparison of food production indicators in developed countries and weakly developed countries.

**Figure 5:**
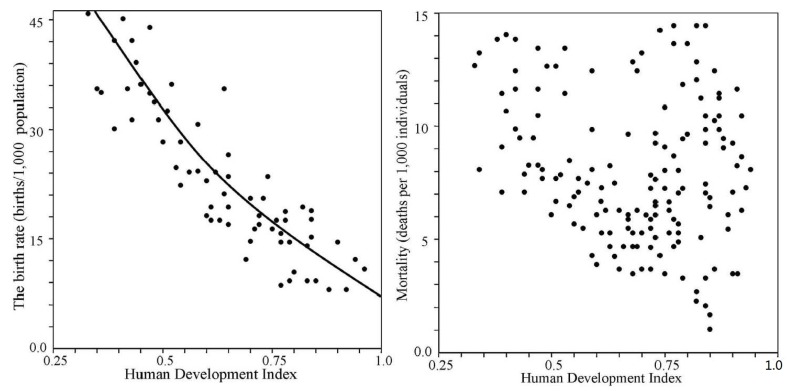
Birth rates and mortality dependencies on the Human Development Index adopted by the countries.

**Figure 6:**
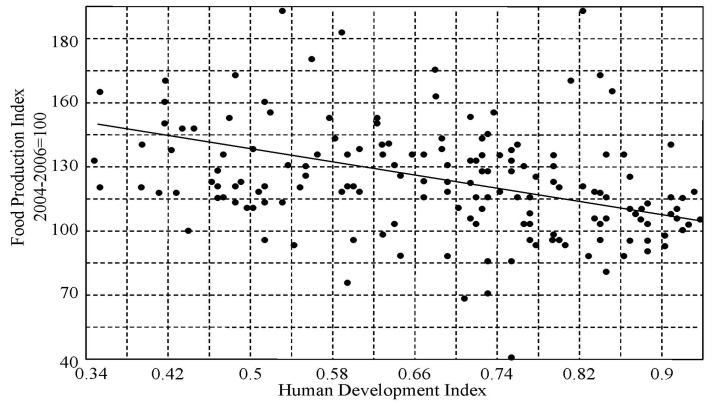
An interdependence of the Food Production Index (FPI) and Human Development Index (HDI) for the different countries.

As can be seen from Figure 2, Figure 3 and Figure 4, the general trend of food production in various countries is characterized by a steady increase in food production. Practically, in the early 21st century, the majority of countries have achieved comparable levels of the food production. However the problem of the food distribution by the individual has not been solved. This problem is quite complex and is connected with socio-economic and culture-ideological area, the parts of which can be distinguished in cardinals depending on the ideology and the traditional conception of the social justice, whose search is carried out with different indicators [72]. According to the results of Figure 5 and Figure 6, the CNSS space indicator has many uncertainties that can be linked to existing causes of non-uniform distribution of vital resources.

Under the premise of peaceful coexistence, the problem of population survivability lies in providing food to those who have to look after the dependencies of global distribution of food and water supplies on the path of globalization.

## 4. Simulation Experiments

**Figure 7:**
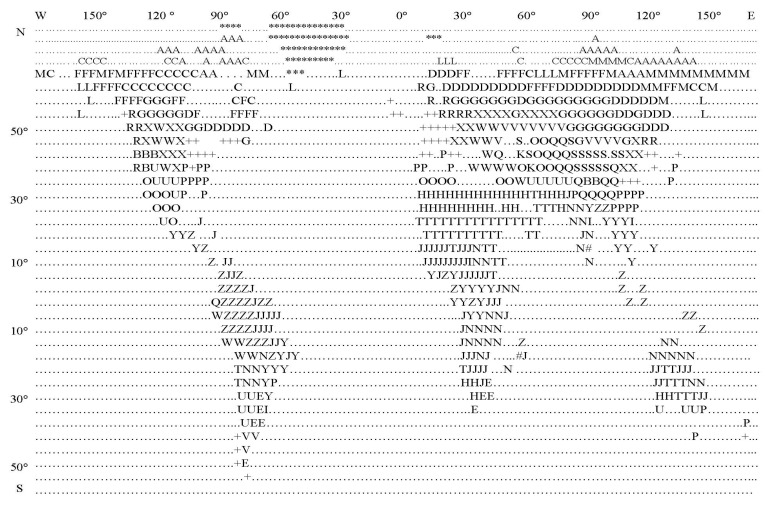
Spatial distribution of the types of soil-plant formations presented in Table 3. Biome indicator is explained in Table 3.

The GIMS/CNSS allows the emulation of different environmental situations using the information and data that define specific characteristics of the land surface, distribution of the soil-plant formations and hydrosphere. The land surface is covered by a discrete number of land cover types depicted in Figure 7 and Table 3. Numerical values of the GIMS/CNSS parameters are given in Table 4. Certainly, these parameters can change over time, but not significantly. Therefore, the parameters of the regions can be interchanged with each other.

It is clear that the accuracy of a forecast can be estimated only after many years or decades. Nevertheless, a complex set of ideas and assumptions in the GIMS/CNSS structure determine a complete picture of the world and form the mechanisms for constructively describing the direct and inverse relationships in which the CNSS survivability is defined by criterion (1).

**Figure 8:**
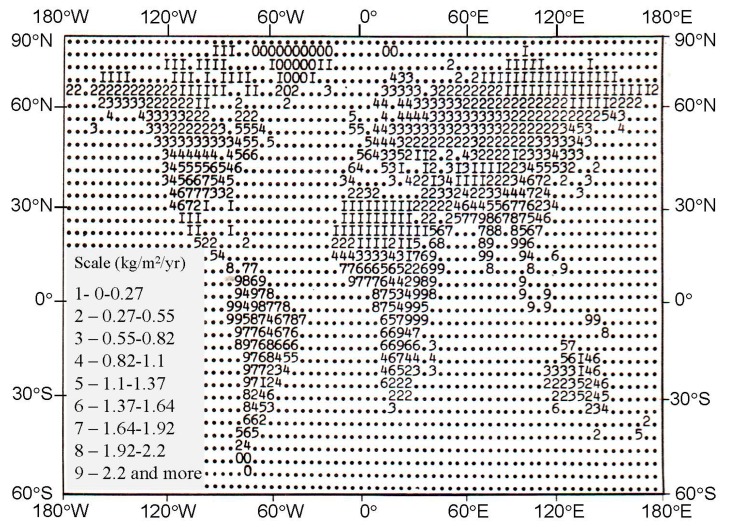
A map-scheme of the productivity of soil-plant formations shown in Table 2 in digital scale with spatial resolution 4° × 5°.

The biocomplexity of the environment precisely determines the level of food supply for the world population. As can be seen from Figure 8, a contribution of nature to this conservation has a non-uniform spatial distribution. The corresponding modern spatial distribution is specific for agriculture and fishery productions.

The GIMS/CNSS items that calculate average regional temperature (CM) and simulate regional hydrological balance (RHCM) allow the estimation of surface vegetation production (item BMSPF) depending on temperature and precipitation (a few estimates are given in Table 5).

It will be assumed that the survivability level *J*(*t*) is the most important for each region. The GIMS/CNSS forms a comprehensive picture of the population dynamics in the pixel structure of the world and taking into account the respective interactions between the biosphere and climatic system. Undoubtedly, the implementation of GIMS/CNSS that is proposed here improves the structure of existing global models and provides more accurately the calculation of the population dynamics.

**Figure 9:**
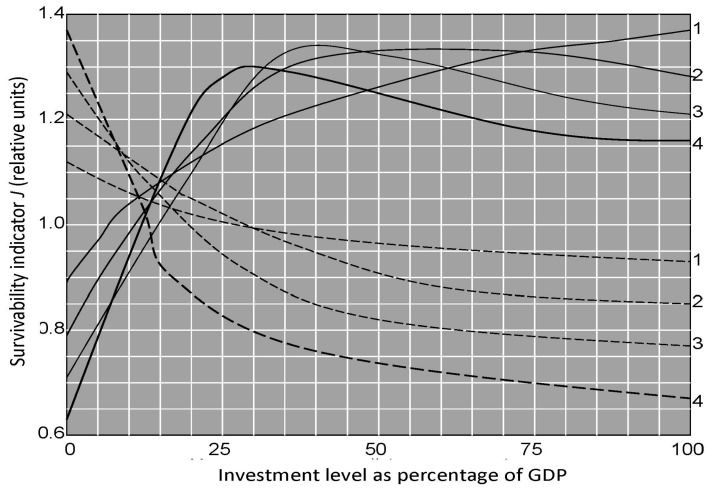
Survivability indicator depending on the GDP distribution by agriculture (solid lines) and industry (dashed lines). Numbers located on the right of the curves show the time periods for the investments: 1–25 years, 2–50 years, 3–75 years, and 4–100 years.

**Figure 10:**
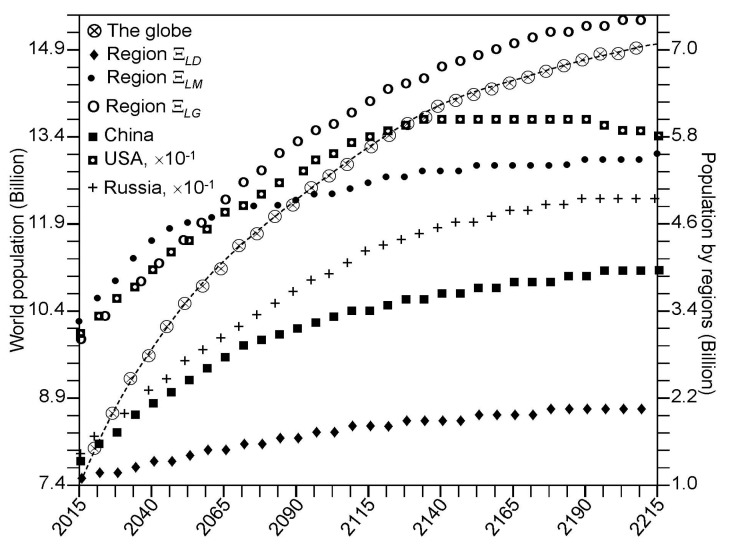
The global and regional population dynamics.

The internal resources for each region are determined by the level of Gross Domestic Product (GDP) and its distribution from the strategic goals. The curves in Figure 9 show the dependence of the system survivability on investment distribution and indicate the level of life of the population according to the distribution of GDP by the economic sectors that is correct over the closest limited time period. Overall, the GIMS/CNSS allows evaluation of the population dynamics under certain assumptions. Let’s look at some of them. Figure 10 represents such evaluations in the context of the following assumptions (scenario SP—scientific progress):

- the problems arising from the limitation of energy sources will be overcome by 2050;
- the emissions of greenhouse gases will increase by 10% by 2050 compared to 2015 and then begin to fall evenly to 2200 up to 5%;
- agricultural technologies to increase productivity by 100% by 2050 and by 200% by the end of the 22nd century will be production;
- the speed of replacement of forest ecosystems by avifauna will be reduced by 10 times in 2050 compared to 2015 and then the forested pixels will not be disturbed; and
- the contribution of World Ocean resources to food production will increase from 1% in 2015 to 5% in 2050 and then increase steadily to 10% in 2200.

**Figure 11:**
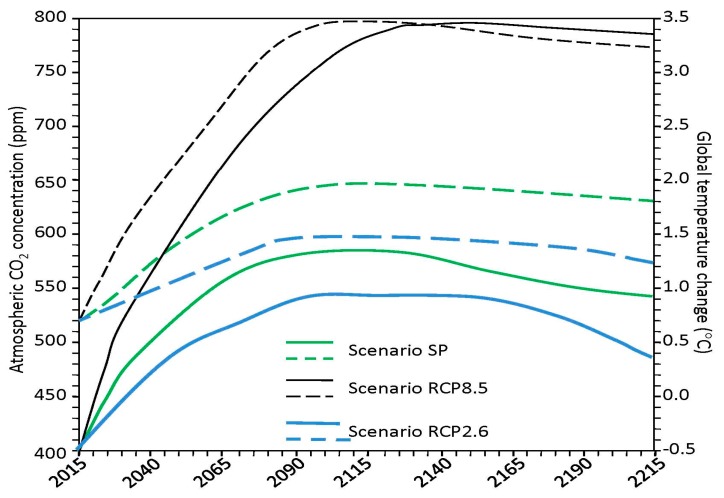
The dynamics of the climatic factors (CO_2_ concentration and temperature are represented by the solid and dashed lines, respectively). A comparison of the results obtained from the implementation of the SP scenario with those from the RCP8.5 and RCP2.6 scenarios [75,76].

As can be seen from the results of Figure 10, population size can reach 14.9 billion at the beginning of 23rd century with a tendency for low growth. The percentage distribution of the population from the regions will change in the direction of the 6.9% increase in the part of the developing countries. Contributions of the regions Ξ*_LD_* and Ξ*_LM_* to population growth declined by 2.1% and 4.8%, respectively. These changes are linked to the different rates of birth and mortality in Equations (6) and (7) as functions of the community status and food supply, as well as climatic parameters. Figure 11 shows some of these characteristics in their dynamics by 2215. It seems that a-priori assumptions about the dynamics of different anthropogenic environmental impacts play an important role in the dynamics of all CNSS components. Unfortunately, these assumptions only occur as specific scenarios.

The implementation of the RCP8.5 scenario (of comparatively high GHS emissions [77]) results in an increase in CO_2_ concentration to 800 ppm in the 23rd century, beginning with the achievement of a maximal surface temperature increase of almost 3 °C. On the other hand, the fairly realistic scenario RCP2.6 (exploring the possibility of maintaining global mean temperature rise below 2 °C [78]) leads to corresponding levels of 520 ppm for CO_2_ and 0.8 °C for temperature change in the middle of 22nd century and after lowering these levels. Therefore, the most accurate forecast requires a detailed analysis by the experts of the current trends in the socio-economic developments of the different regions. However, even these hypothetical scenarios provide information to think about the possible safe ways of population growth when survivability is maintained for a long time.

**Figure 12:**
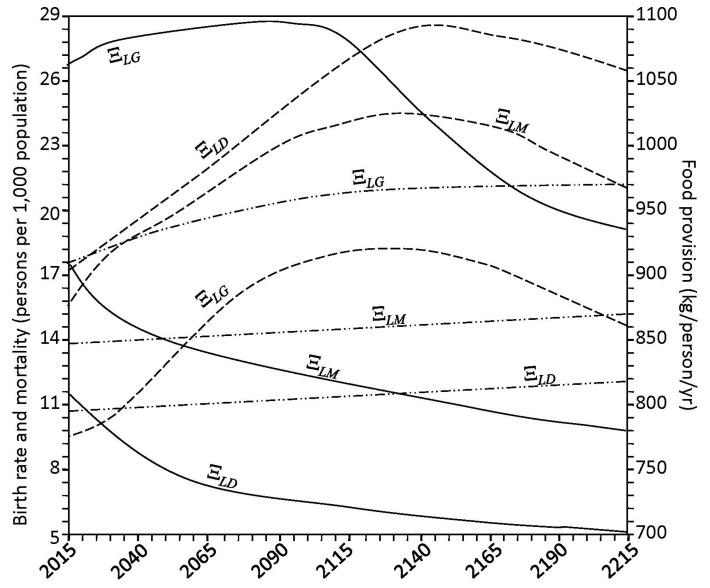
The dynamics of the vital factors (supply of food by region, birth rate, and mortality are represented by the broken, solid, and chain lines, respectively). Regional identifiers are placed on the curves.

Figure 12 shows a dynamics of the key factors that are linked with evolution process of the society development. The birth rate coefficients μ*_B_* for the Ξ*_LD_*, Ξ*_LM_* and Ξ*_LG_* regions are change from 0.0115, 0.0177 and 0.0267 in 2015 to 0.005, 0.0098 and 0.0191 in 2200, respectively. According to this, the birth rate coefficients of the Ξ*_LD_* and Ξ*_LM_* regions will decrease evenly with time, and the birth rate coefficient will reach the maximal value 0.034 in the Ξ*_LG_* region in 2060 and then decrease. The mortality coefficients μ*_d_* are similarly modified in the Ξ*_LD_*, Ξ*_LM_* and Ξ*_LG_* regions from 0.0107, 0.0138 and 0.0175 in 2015, to 0.0121, 0.0153 and 0.0211 in 2200, respectively.

## 5. Conclusions

The proposed version of the global geo-ecological information-modeling system provides tools for studying and evaluating the limiting anthropogenic impacts on the biosphere and allows for the understanding of its responses and identification of the exclusion area for possible human activity. In this context, the GIMS/CNSS provides the capability to detect regional ecological responses to the effects identified in the limited number of spatial pixels. The GIMS/CNSS is based on combined use of specific models of particular environmental processes listed in Table 1 and tested separately. The parameters of the model such as ρ, *β*, χ_1_, χ_2_, χ_3_, ξ_1_, ξ_2_ and ξ_3_ are corrected, based on the minimal discrepancy between modeling results and the prehistory of trends in the global population and atmospheric CO_2_ during 2000–2015. The model verification is based on a comparison of the prehistory trends of the real global temperature with those deduced by the model. In this case the average deviation for the period 2000–2015 was no more 7%.

The GIMS/CNSS can be used to evaluate the consequences of the implementation of anthropogenic scenarios, such as spatial reconstruction of soil-plant formations or changes in the vegetation cover as a result of wildfires. The modelled changes are accomplished by replacing literal symbols in the map of the soil-plant formations (Figure 7). Preliminary calculations have shown a strong dependence of the CO_2_ cycle [79,80] on changes in vegetation cover.

Undoubtedly, the GIMS/CNSS reflects the limited range of feedbacks in CNSS with emphasis on ecological interactions. The GIMS/CNSS allows the modernization its structure through additional items that shape the socio-economic and living feedbacks in the global climate system.

It should be noted that the GIMS/CNSS Global Model developed here is not comparable to other available complex global models. The model of global environmental processes based on the GIMS-technology differs largely from other global models from the ability to evolutionary adapt to pre-history using informative indicators on the state of CNSS. Certainly, the adaptation process and the selection of informative indicators are needed in the additional surveys.

The results of this study show that survivability problem will not be critical over the next two centuries, depending on the population growth. Restrictions on the availability of food production resources will occur at the end of 21st century when, as shown in Figure 12, the global nuclear power plant (NPP) is slowly declining due to climate change and changes in regional hydrological balances. In particular, the rise in temperature in tropical latitudes causes a decrease in water content in the soil due to the evaporation which leads to the NPP decrease. In contrast, in northern pixels, the rise in temperature leads to a 16–20 day extension in the 22nd century, starting with a 9–12% increase in the NPP. These negative and positive feedbacks are not evenly distributed by the pixels. As a result, the food production dynamics illustrated in Figure 12 shows that the export of excess of food stocks of the region Ξ*_LG_* to other regions is only possible until the end of 21st century, as the human population expands the effectiveness of such strategies, such as expansion of the land area used for agriculture, the expansion of fishing, and the increase in agricultural productivity. Current trends in increasing the regional population suggest that satisfying food demands is unlikely to occur if human society does not seek sustainable interactions with nature. Realized food production estimates are approximate and can be more accurate when spatial digitization of land and oceans will be, for example, 0.5° × 0.5° or less. It is known that changes in net primary production in the ocean vary from 1800 g/m^2^/year in estuaries to 50 g/m^2^/year in the open ocean. Biomass variations and biomass production of the land vegetation have a wide range as well. This circumstance is an additional reserve to make the results of the global model more accurate. Certainly, the GIMS/CNSS model allows for a more detailed description of the soil-plant formations depicted in Figure 7 taking into account the existing site variations and productivity, as well as the specifications of agricultural ecosystems. Additional enlargement and identification of global and regional environmental databases are required. Furthermore, the analysis presented showed that all the given assumptions are closely related to the results of the presented model. In addition, the potential use of the presented model at regional and global level is presented in [22,23,46,47]. It would be of particular interest to apply this model to investigate the impact on public health from modern environmental problems, such as the depletion of the ozone layer and the induced increase in solar ultraviolet radiation reaching the ground [81,82,83,84,85,86,87].

## Figures and Tables

**Table 1:** The GIMS/CNSS functional items.

Item	Item Functions
DM	Demographic model [53].
CM	Climate model [42,54].
CMCM	Coupled model of the carbon dioxide and methane cycles [47].
GSCM	Global sulphur cycle model [22].
GCOO	Coupled model of global cycles of oxygen and ozone [22].
GNCM	Global nitrogen cycle model [55].
GPCM	Global phosphorus cycle model [56].
RHCM	Regional hydrological cycle model [57].
BMSPF	Biocenotic model of the soil-plant formations [48,50].
PMTM	Photosynthesis model for the tropical and moderate oceanic zones [58].
PMAA	Photosynthesis model for the Arctic and Antarctic zones of the World Ocean [19,59,60].
APM	Agriculture production model [61,62].
AIFI	Evolutionary algorithm for the indicator calculation of the food industry [44,50].
UEM	An upwelling ecosystem model [46].
MWEL	Model of the typical water ecosystem on the land [57].
AHIS	An algorithm for the human indicator survivability calculation.
DMEP	Dynamic model of the environmental pollutants [56].
GSA	The GIMS structure adaptation to the simulation experiment conditions [23,42].
DFM	Database formation and management.
SS	Synthesis of the scenarios for the interaction of population with the environment.
SEMC	Simulation experiment management and control.

**Table 2:** List of the model parameters.

Parameter	Symbol	Parameter Evaluation
Photosynthesis compensation constant:	*Γ*, ppmv	
Equator		5
Pole		50
Coefficient reflecting the effect of the CO_2_ factor on plant production.	*a_C_*	3.226
Constant of the photosynthetic responses to atmospheric CO_2_ changes.	*b_C_*, ppmv	930.03
Coefficient reflecting the impact of solar radiation on plant production.	*a_E_*	1.177
Parameter indicating the solar radiation in which the stability of plant production is achieved.	*b_E_*, W/m^2^	60.538
Coefficient reflecting the effect of precipitation on plant production.	*a_W_*	4.742
Parameter indicating the precipitation in which the stability of plant production is achieved.	*b_W_* mm/year	592.357
Parameter indicating the maximal rate of growth of plant biomass under temperature change.	*a_T_*	0.56
Indicator of declining plant production under temperature change.	*b_T_*	0.42
Maximal rate of loss of plant biomass under temperature change.	*ρ_T_*	1.214
Parameter that controls early delay to achieve a maximal rate of loss of plant biomass due to temperature change.	*d_T_*, °C	5.714
Maximal rate of loss of plant biomass under change of soil moisture.	*d_a_*	0.0267
Parameter that controls early delay to achieve a maximal rate of loss of plant biomass to precipitation change.	*d_b_*, mm/year	208.333
Ratio coefficient that characterizes phytoplankton rate dependence on temperature.	*θ_W_*	0.21
Ratio coefficient that characterizes phytoplankton rate dependence on solar energy.	*θ_E_*	0.25
Constant that determines the characteristics of phytoplankton species dependent on biogenic salts.	*γ_N_*	0.1
Constant that determines phytoplankton production as a function of its biomass.	*γ_P_*	0.25
The area of the biosphere.	*σ*, km^2^	510.1 × 10^6^
Model start time.	*t*_0_	2015

**Table 3:** Quantitative characteristics of the formation types of the land vegetation [60,63,73,74]. *Notation:* σ*_S_* is the biome area (mln km^2^), $R_{\Phi }^{*1}$ is the annual increment of plants (kg/m^2^/year), Φ* is the phytomass (kg/m^2^). *T*_min_ and *T*_opt_ are minimal and optimal temperatures for photosynthesis, respectively.

Indicator and Type of Soil-Plant Formation	*σ_S_*	$R_{\Phi }^{*1}$	Φ*	*T*_min_ (°C)	*T*_opt_ (°C)
*A—*Arctic deserts and tundra	2.55	0.17	0.4	−5	40
*C—*Tundra	2.93	0.36	1.9	−5	40
*M—*Mountain tundra	2.33	0.38	1.9	−3	35
*L—*Forest tundra	1.55	0.65	3.8	−5	40
*F—*North-taiga forests	5.45	0.54	10	−5	40
*D—*Mid-taiga forests	5.73	0.63	22.5	−5	40
	6.6	0.65	23.5	−5	40
*G—*South-taiga forests	2.12	0.87	25	−1	43
	7.21	1.25	45	−1	43
*R—*Broad-leaved coniferous forests	5.75	1.72	43	0	43
+*—*Broad-leaved forests	3.91	0.56	3.8	0	43
*P—*Sub-tropical broad-leaved and coniferous forests	3.72	0.74	1.9	2	43
*U—*Xerophytic open woodlands and shrubs	4.29	0.79	1.9	2	43
*X—*Forest-steppes (meadow steppes)	1.66	1.11	3.8	5	45
*W—*Moderately arid and arid (mountain including) steppes	2.66	0.38	0.8	5	45
*E—*Pampas and grass savannas	2.08	0.45	0.4	5	45
*V—*Dry steppes	2.69	0.25	0.2	5	50
#*—*Mangrove forests	1.99	0.35	0.8	5	30
*S—*Sub-boreal and saltwort deserts	7.16	0.12	0.1	5	45
&*—*Sub-tropical semi-deserts	1.15	0.47	0.8	−3	10
*H—*Sub-tropical deserts	3.54	0.76	1.9	−3	10
*B—*Alpine deserts	10.4	3.17	60	5	50
*Q—*Alpine and sub-alpine meadows	7.81	2.46	60	5	50
*Z—*Humid evergreen tropical forests	9.18	1.42	10	5	50
*Y—*Variably-humid deciduous tropical forests	17.1	1.35	0.1	5	45
*N—*Tropical xerophytic open woodlands	13.52	0.18	0.4	5	45
*J—*Tropical savannas	0.38	0.18	45	4	50
*T—*Tropical deserts	0.9	1.96	45	4	45
*K—*Saline lands	14.6	0	0	-	-
*I—*Sub-tropical & tropical grass-tree thickets of the tugai type					
*—Lack of vegetation					

**Table 4:** Coefficients of the GIMS/CNSS for the land surface.

Coefficient	Region Ξ*_LD_*	Region Ξ*_LM_*	Region Ξ*_LG_*
ρ, year^−1^	1.19	1.26	1.32
*β*, year^−1^	1.21	1.23	1.25
η_1_	0.01	0.011	0.014
η_2_	0.003	0.005	0.009
ξ_1_	0.031	0.027	0.025
ξ_2_	0.012	0.011	0.009
ξ_3_	0.006	0.005	0.004
χ_1_	0.035	0.032	0.031
χ_2_	0.014	0.012	0.011
χ_3_	0.003	0.002	0.001
μ_1_	0.02	0.03	0.04
μ_2_	0.005	0.009	0.012
*γ*, °C	34	34	34
ϖ	0.56	0.61	0.67

**Table 5:** The dependence of the annual vegetation production *R_Φ_*(*T_Ξ_*,*W_Ξ_*) (kg/m^2^/year) on the average annual temperature (*T_Ξ_*) and full precipitation (*W_Ξ_*).

Precipitation, *W_Ξ_* (mm/Year)	Atmospheric Temperature, *T_Ξ_* (°C)											
	−14	−10	−6	−2	2	6	10	14	18	22	26	30
3130							3.39	3.49	3.68	3.81	3.92	4.01
2880							3.27	3.36	3.47	3.63	3.73	3.82
2630							3.09	3.27	3.31	3.44	3.54	3.65
2380							2.85	2.93	3.09	3.12	3.22	3.33
2130							2.57	2.69	2.67	2.94	2.91	3.03
1880						1.63	2.38	2.38	2.43	2.55	2.62	2.74
1630				0.39	0.62	1.34	2.04	2.14	2.12	2.26	2.35	2.42
1380		0.18	0.31	0.41	0.73	1.16	1.75	1.91	1.95	2.13	2.18	2.09
1130	0.19	0.26	0.32	0.43	0.77	1.05	1.66	1.84	1.92	1.84	1.83	1.75
880	0.21	0.28	0.42	0.52	0.83	0.92	1.53	1.43	1.33	1.36	1.27	1.24
630	0.28	0.29	0.53	0.57	0.89	0.91	0.92	0.85	0.84	0.73	0.72	0.71
380	0.39	0.41	0.54	0.69	0.66	0.64	0.67	0.57	0.56	0.55	0.43	0.42
130	0.14	0.32	0.31	0.22	0.24	0.24	0.24	0.24	0.23	0.14	0.13	0.11
